# Relationship between porcine miR-20a and its putative target low-density lipoprotein receptor based on dual luciferase reporter gene assays

**DOI:** 10.5713/ajas.18.0510

**Published:** 2019-01-03

**Authors:** Yueyun Ding, Shujiao Zhu, Chaodong Wu, Li Qian, DengTao Li, Li Wang, Yuanlang Wang, Wei Zhang, Min Yang, Jian Ding, Xudong Wu, Xiaodong Zhang, Yafei Gao, Zongjun Yin

**Affiliations:** 1 Anhui Provincial Laboratory of Local Animal Genetic Resource Conservation and Bio-Breeding, College of Animal Science and Technology, Anhui Agricultural University, Hefei, Anhui 230036, China; 2 Anhui Haoxiang Agriculture And Animal Husbandry Co. LTD, Bozhou, Anhui 236700, China

**Keywords:** *SSC*-miRNA-20a, Low-density Lipoprotein Receptor (LDLR), pGL3-Control Vector, pMR-mCherry Vector, Dual Luciferase Reporter Gene Assay System

## Abstract

**Objective:**

Mutations in low-density lipoprotein receptor (*LDLR*), which encodes a critical protein for cholesterol homeostasis and lipid metabolism in mammals, are involved in cardiometabolic diseases, such as familial hypercholesterolemia in pigs. Whereas microRNAs (miRNAs) can control *LDLR* regulation, their involvement in circulating cholesterol and lipid levels with respect to cardiometabolic diseases in pigs is unclear. We aimed to identify and analyze *LDLR* as a potential target gene of *SSC*-miR-20a.

**Methods:**

Bioinformatic analysis predicted that porcine *LDLR* is a target of *SSC*-miR-20a. Wild-type and mutant *LDLR* 3′-untranslated region (UTR) fragments were generated by polymerase chain reaction (PCR) and cloned into the pGL3-Control vector to construct pGL3 Control LDLR wild-3′-UTR and pGL3 Control LDLR mutant-3*′*-UTR recombinant plasmids, respectively. An miR-20a expression plasmid was constructed by inserting the porcine pre-miR-20a-coding sequence between the *Hind*III and *Bam*HI sites in pMR-mCherry, and constructs were confirmed by sequencing. HEK293T cells were co-transfected with the miR-20a expression or pMR-mCherry control plasmids and constructs harboring the corresponding 3′-UTR, and relative luciferase activity was determined. The relative expression levels of miR-20a and LDLR mRNA and their correlation in terms of expression levels in porcine liver tissue were analyzed using reverse-transcription quantitative PCR.

**Results:**

Gel electrophoresis and sequencing showed that target gene fragments were successfully cloned, and the three recombinant vectors were successfully constructed. Compared to pMR-mCherry, the miR-20a expression vector significantly inhibited wild-type LDLR-3′-UTR-driven (p<0.01), but not mutant LDLR-3′-UTR-driven (p>0.05), luciferase reporter activity. Further, miR-20a and *LDLR* were expressed at relatively high levels in porcine liver tissues. Pearson correlation analysis revealed that porcine liver miR-20a and *LDLR* levels were significantly negatively correlated (r = −0.656, p<0.05).

**Conclusion:**

*LDLR* is a potential target of miR-20a, which might directly bind the *LDLR* 3′-UTR to post-transcriptionally inhibit expression. These results have implications in understanding the pathogenesis and progression of porcine cardiovascular diseases.

## INTRODUCTION

Serum lipids, including total cholesterol, low-density lipoprotein cholesterol (LDL-C), high-density lipoprotein cholesterol, and triglycerides, are important clinical diagnostic indices of cardiovascular diseases [1]. Despite progress in the treatment of abnormal levels of cholesterol and other lipids, cardiometabolic diseases represent prominent causes of human morbidity and mortality [2]. Pigs are widely used in biomedical research [3,4]. As their heart size, blood supply, coronary system function, and aorta features are comparable to those in humans, the pig is an important model for cardiovascular disease research [5]. Several candidate genes for porcine serum lipids, such as low-density lipoprotein receptor (*LDLR*), apolipoprotein B, and low density lipoprotein receptor adapter protein 1 (*LDLRAP1*), have been reported to date [6]. The LDLR is a cell membrane glycoprotein that plays a critical role in cholesterol homeostasis and lipid metabolism in mammals [7]. Further, *LDLR* gene mutations play a major role in the progression of cardiometabolic diseases [8]. In pigs, loss-of-function mutations in this gene result in the accumulation of LDL-C in circulation, leading to familial hypercholesterolemia [9], a severe disease characterized by premature death due to accelerated cardiovascular disease. LDL-C uptake by the LDLR in the liver is of clinical importance as LDLR-dependent hepatic clearance of circulating LDL-C has a prominent role in preventing atherosclerosis and cardiovascular disease [10]. The recent finding that manipulating miRNA expression might reduce circulating LDL-C could lead to a new option for the treatment of dyslipidemias [11].

miRNAs, comprising a class of short (20 to 24 nt) regulatory RNAs that modulate mRNA translation and turnover, have recently emerged as crucial regulators of mammalian cholesterol and lipid homeostasis [12]. Several miRNAs have been found to control aspects of cholesterol and lipid homeostasis at the cellular level and *in vivo* [13]. Specifically, recent work uncovered a role for miRNAs in directly controlling the LDLR activity *in vitro* and *in vivo* by post-transcriptionally regulating the *LDLR* gene [14]. This miRNA-centered regulatory network continues to grow rapidly and will undoubtedly expand to include other miRNAs that regulate LDL-C levels. Future studies using non-human primates will likely help determine the relative contribution of each miRNA in controlling LDLR activity and cholesterol homeostasis [15]. However, the potential contribution of miRNAs to dysregulated circulating cholesterol and lipids and associated cardiometabolic diseases in pigs remains unclear.

Because of the physiological and clinical importance of the LDLR in regulating circulating LDL-C, and as porcine *LDLR* expression was not previously shown to be under miRNA control, we conducted bioinformatic analysis to predict miRNAs that could bind the porcine *LDLR* 3′-untranslated region (UTR). We found that *Sus scrofa* (*SSC*)-miR-20a could directly bind *LDLR* mRNA with low binding free energy. Next, we constructed an *SSC*-LDLR 3′-UTR dual luciferase reporter vector, a vector encoding the 3′-UTR mutated at the miR-20a-binding site, and an *SSC*-miRNA-20a expression plasmid. A dual luciferase assay system was used to detect relative luciferase activities. Additionally, we assessed the correlation between miR-20a and *LDLR* expression levels in porcine liver tissues. This study aimed to provide a theoretical basis for elucidating the direct relationship between *SSC*-miR-20a and *LDLR*, as well as the possible mechanism through which miR-20a and LDLR prevent dyslipidemias and cardiovascular diseases.

## MATERIALS AND METHODS

### Ethics statement

All animal experiments were conducted according to the Regulations and Guidelines for Experimental Animals established by the Ministry of Science and Technology (Beijing, China, revised in 2004) and approved by the Institutional Animal Care and Use Committee of Anhui Agricultural University (Permit number: SYXK 2016-007).

### Materials

Human embryonic kidney (HEK) 293T cells were cultured in high-glucose Dulbecco’s modified Eagle’s medium (Invitrogen Inc., Carlsbad, CA, USA) containing 10% fetal bovine serum. The cells were maintained at 37°C in the presence of 5% CO_2_. Liver, heart, spleen, lung, and kidney tissues from 12 Large White castrated boars of approximately 100 kg were collected. Tissues were frozen in liquid nitrogen immediately after resection and stored at −80°C until use.

### Bioinformatic analysis

We retrieved miRNAs from the miRBase database (http://www.mirbase.org/) that might associate with the SSC-LDLR 3′-UTR using miRanda (http://www.microrna.org), miRDB (http://www.mirdb.org/mirdb/policy.html), and Targetscan (http://www.Targetscan.org) software. Based on predictive criteria, including having good complementarity with targeted sequences, being bound to targeted sequences with low free energy of binding, and being conserved among species, miR-20a was selected as a candidate miRNA for follow-up studies.

### Reverse transcription quantitative polymerase chain reaction

Total RNA was extracted from porcine liver tissues using TRIzol reagent (Invitrogen; Thermo Fisher Scientific, Inc., Waltham, MA, USA), according to the manufacturer’s protocol. RNA concentrations and purity were measured with an ND 2000 spectrophotometer (NanoDrop; Thermo Fisher Scientific, Inc., Wilmington, DE, USA).

For the quantitative analysis of miR-20a expression, 1 μg of total RNA from each sample was reverse-transcribed into cDNA using the NCode EXPRESS SYBR GreenER miRNA qPCR Kit (Invitrogen, USA) at 37°C for 1 h and 85°C for 5 s, in a 20-μL reaction mixture, according to the manufacturer’s instructions. With reference to miRBase 21.0 (http://www.mirbase.org/), we designed miRNA-specific forward primers by poly (A)-tailing miR-20a; the universal reverse primer was provided in the Invitrogen kit. Porcine U6 small nuclear RNA (U6) was used as an endogenous control gene.

For quantitative analysis of *LDLR* mRNA expression, 1 μg of total RNA from each sample was reverse-transcribed into cDNA using the Prime Script RT Kit (TaKaRa, Tokyo, Japan) at 37°C for 15 min and 85°C for 5 s in a 10-μL reaction mixture, according to the manufacturer’s instructions. The *β-actin* gene was used as an endogenous control gene.

All primers used are listed in Table 1. Quantitative polymerase chain reaction (qPCR) was performed in triplicate using a CFX96 Touch real-time PCR detection system (Bio-Rad, Hercules, CA, USA). Thermal cycling conditions were as follows: 94°C for 30 s, followed by 40 cycles at 94°C for 5 s, and 60°C for 30 s. The relative expression of miR 20a and *LDLR* mRNA was determined using the 2^−ΔΔCt^ method [16].

### Plasmid construction

**Figure 1:**
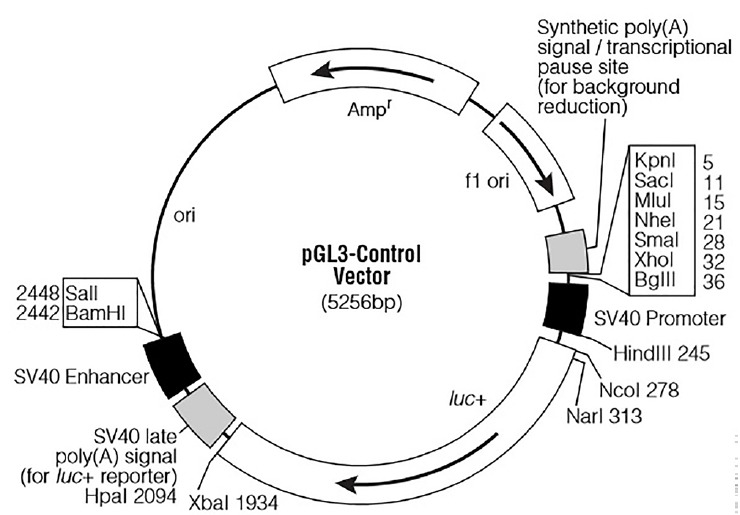
pGL3-Control vector map.

For the functional analysis of miR-20a, a partial segment of the *LDLR* mRNA 3′-UTR containing the miR-20a-binding sequence and a mutated segment of the *LDLR* mRNA 3′-UTR in which the miR-20a-binding sequence AAGCACTG was converted to AACGACTG, were PCR amplified from DNA prepared from porcine liver tissues. Based on the porcine *LDLR* mRNA 3′-UTR sequence (NCBI: NM_001206354.2), primer sequences were designed (Table 2). The expected length of both amplicons was 688 bp. The PCR products were subcloned into the *Xba*I site downstream of the stop codon in the PGL3-Control firefly luciferase reporter vector (Promega, Madison, WI, USA; the vector backbone is shown in Figure 1). Recombinants harboring the desired PCR products were confirmed by DNA sequencing. The recombinant plasmids were termed “WT” (pGL3 Control LDLR wild-3′-UTR) and “MT” (pGL3 Control LDLR mutant -3′-UTR).

**Figure 2:**
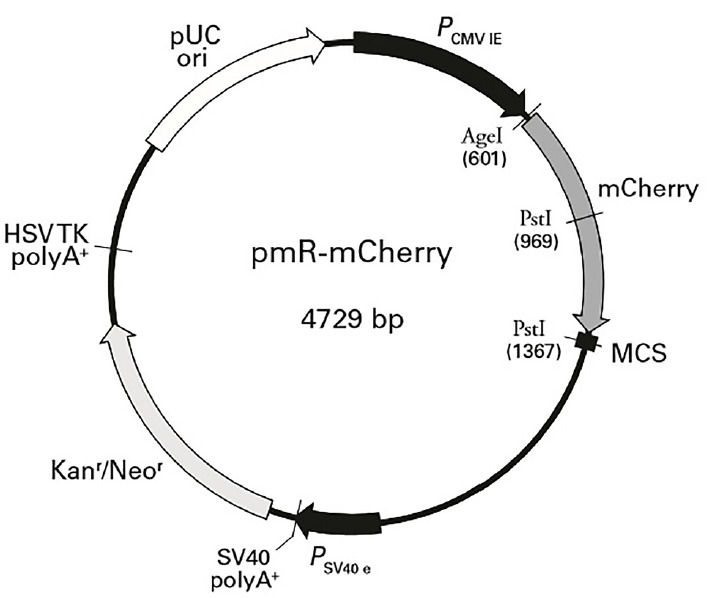
pmR-mCherry vector map. MCS, multiple cloning site.

Pre-miR-20a coding sequences were PCR-amplified from cDNA prepared from porcine liver tissues. Primer 5.0 was used to design PCR primers based on the sequence of porcine miR-20a (NCBI: MIMAT0002129). Primer sequences are shown in Table 2. The expected length of the amplicon was 170 bp. MiR-20a expression plasmids were constructed by inserting a DNA fragment containing the pre-miRNA coding sequence between the *Hind*III and *Bam*HI (Fermentas, Vilnius, Lithuania) sites of pMR-mCherry (Clontech, Palo Alto, CA, USA; the vector backbone is shown in Figure 2). The constructs containing the pre-miRNA coding sequences were confirmed by sequencing and termed “miR-20a expression vectors”.

### Plasmid transfection and luciferase assays

Plasmids were transfected using Lipofectamine 2000 (Invitrogen, USA) according to the supplier’s protocol. For luciferase assays, HEK293T cells at 70% confluence were transferred into 24-well plates with miR-20a expression plasmids or the pMR-mCherry control plasmid, as well as the firefly luciferase constructs containing the corresponding 3′-UTR. Luciferase activity was measured 24 h post-transfection using the Dual-Luciferase Reporter 1000 System (Promega, USA), based on the manufacturer’s protocol. Briefly, cells were lysed with passive lysis buffer at room temperature for 15 min. Luciferase assay buffer II was then added, and firefly luciferase activity was immediately determined using a Fluoroskan Ascent FL microplate reader (Thermo Scientific, Waltham, MA, USA). Next, Stop & Glo Buffers with Stop & Glo substrates were added and mixed briefly. *Renilla* luciferase activity was immediately determined and firefly luciferase activity was normalized to *Renilla* luciferase activity to account for variations in transfection efficiency. All reactions were performed in triplicate.

### Statistical analysis

Data are expressed as the mean±standard error of the mean. A Student’s t-test was applied to compare two groups and one-way analysis of variance was performed for multiple groups; the association between liver miR-20a and *LDLR* mRNA expression levels was analyzed by calculating the Pearson’s correlation coefficient. Analyses were conducted with SPSS software (version 19.0; IBM, Armonk, NY, USA). Less than 0.05 p value was considered to indicate a statistically significant difference and less than 0.01 p value was considered to indicate an extremely statistically significant difference.

## RESULTS

### Target prediction

**Figure 3:**
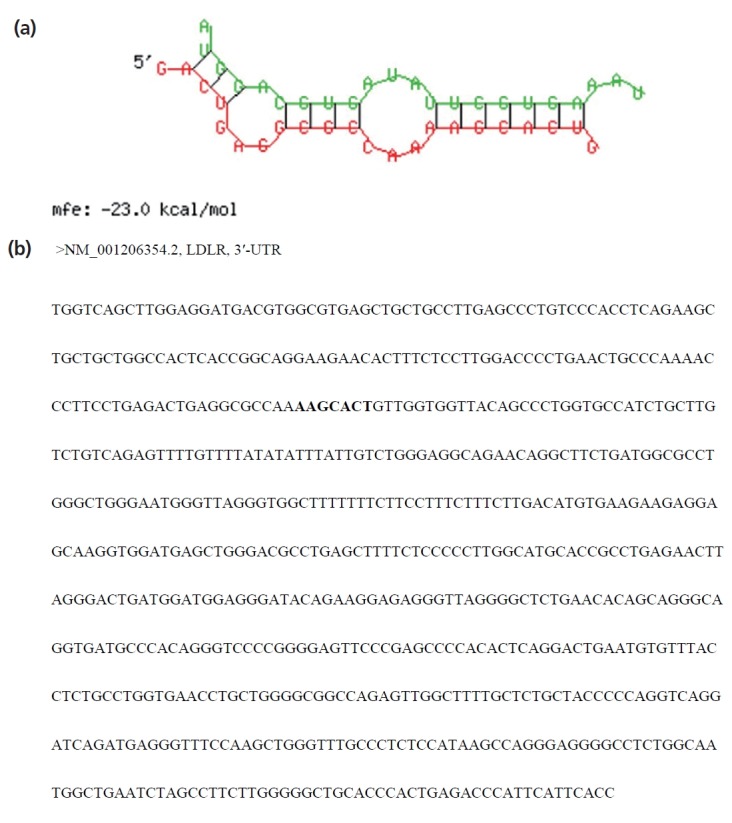
Target prediction results of porcine *LDLR* 3′-UTR binding. (a) Comparison of base-pair stem-and-loop structure between mature *SSC*-miR-20a and *LDLR* 3′-UTR. (b) A potential target site for *SSC*-miR-20a was found to be located in the 3′-UTR of the *LDLR* gene. *LDLR*, low-density lipoprotein receptor; UTR, untranslated region.

Using miRanda, TargetScan, and miRDB programs, *LDLR* was predicted as a potential target of miR-20a (Figure 3). As shown in Figure 3a, the *SSC*-miR-20a and *LDLR* 3′-UTR sequences were determined to have good complementarity and to bind each other with low binding free energy (approximately −23.0 kcal/mol). As shown in Figure 3b, one potential target site for *SSC*-miR-20a was identified in the *LDLR* 3′-UTR. These results suggested that *LDLR* is a target of *SSC*-miR-20a.

### Identification of polymerase chain reaction products by agarose gel electrophoresis

**Figure 4:**
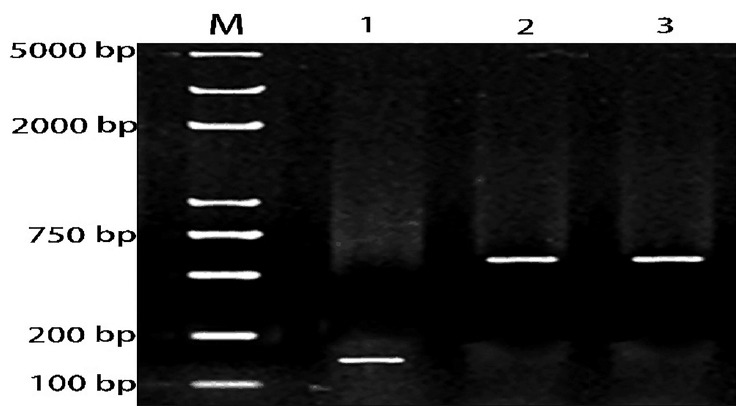
Agarose gel electrophoresis analysis of PCR products. M: DNA marker; lane 1: PCR product of miR-20a precursor; lane 2: PCR product of *LDLR* mRNA 3′-UTR sequence; lane 3: PCR product of *LDLR* mRNA 3′-UTR mutant sequence. PCR, polymerase chain reaction; *LDLR*, low-density lipoprotein receptor; UTR, untranslated region.

A partial segment of the *LDLR* mRNA 3′-UTR containing the miR-20a-binding sequence and a mutated segment of the *LDLR* mRNA 3′-UTR in which the miR-20a-binding sequence AAGCACTG was converted to AACGACTG, were PCR amplified from DNA prepared from porcine liver tissues. Gel electrophoresis results showed that the PCR products were specific and of the expected size (Figure 4).

### Confirmation of plasmid construction

**Figure 5:**
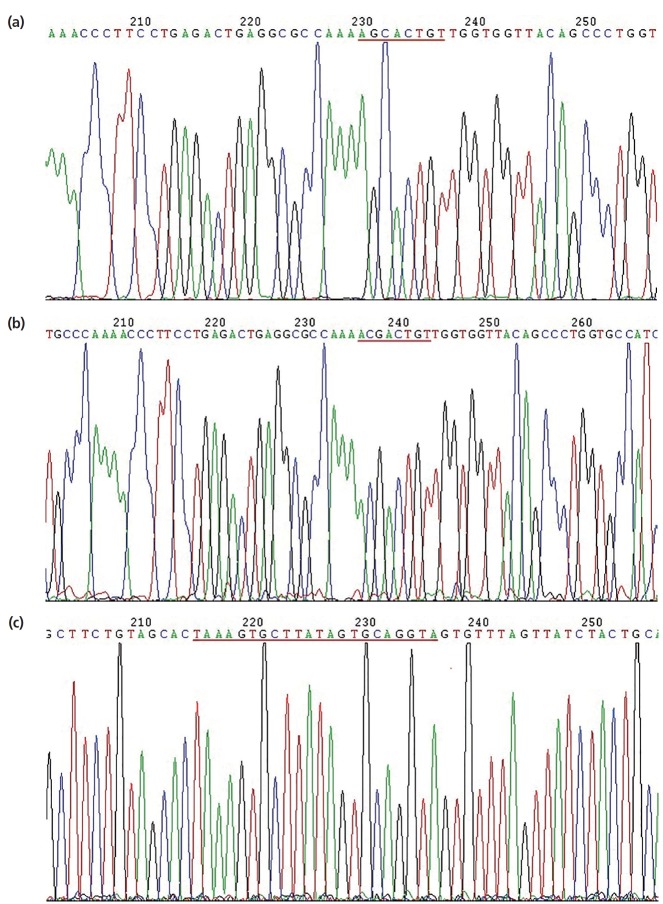
Sequencing results of recombinant plasmids. (a) Sequencing results of dual luciferase reporter vector containing the *LDLR* 3′-UTR; (b) sequencing results of dual luciferase reporter vector containing the *LDLR* 3′-UTR with a target sequence mutation; (c) sequencing results of miR-20a expression vector. *LDLR*, low-density lipoprotein receptor; UTR, untranslated region.

Sequencing results of the pGL3-Control-LDLR-wild-3′-UTR vector are shown in Figure 5a; the cloned fragment had 100% sequence identity with the corresponding region of the porcine *LDLR* gene listed in GenBank. Sequencing results of the pGL3-Control-LDLR-mutant-3′-UTR vector are shown in Figure 5b; the AAGCACT sequence was successfully mutated to AACG ACT, without changes to other bases. These results confirmed that LDLR-wild-3′-UTR and LDLR-mutant-3′-UTR were successfully cloned into the dual luciferase reporter vectors. Sequencing results of the miR-20a expression vector are shown in Figure 5c; the sequence of the miR-20a fragment was in good agreement with the designed pre-miR-20a coding sequence, indicating that the miR-20a expression vector was successfully constructed.

### Role of miR-20a in regulating low-density lipoprotein receptor 3′-UTR

**Figure 6:**
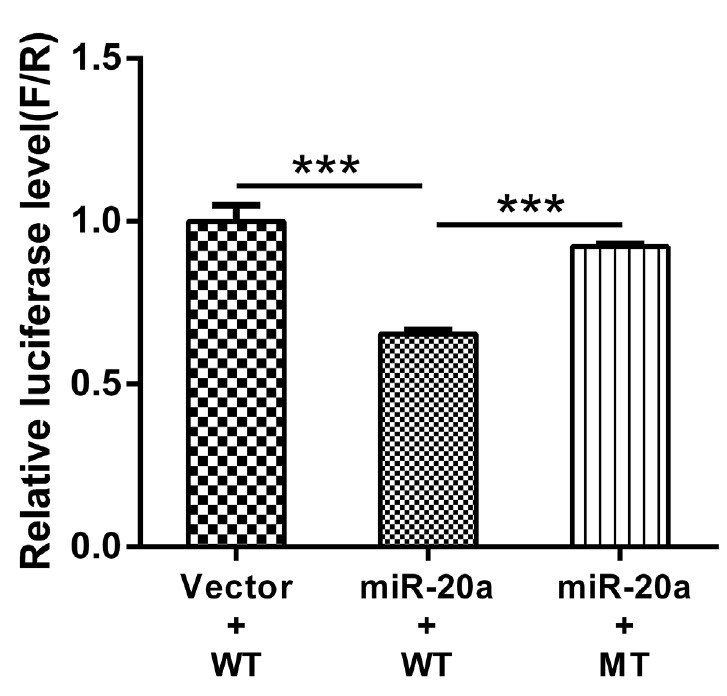
Luciferase activity in HEK293T cells transfected with reporter vectors containing *LDLR* mRNA 3′-UTR variants and an miR-20a expression vector. HEK293T, human embryonic kidney 293T; *LDLR*, low-density lipoprotein receptor; UTR, untranslated region; WT, wild-type LDLR 3′-UTR; MT, LDLR 3′-UTR with a target sequence mutation in miR-20a-binding site. Error bars represent the standard error of three independent experiments. *** p<0.01.

Firefly luciferase activities in HEK293T cells transfected with the recombinant plasmids are shown in Figure 6. Compared to the pMR-mCherry control, the miR-20a expression vector significantly inhibited wild-type LDLR-3′-UTR activity (p< 0.01). However, the miR-20a expression vector had no effect on luciferase activity from the *LDLR* mRNA 3′-UTR reporter containing the mutated miR-20a-binding site. These results indicated that miR-20a targets *LDLR* via the miR-20a-binding site located in the *LDLR* mRNA 3′-UTR and inhibits its transcriptional activity, which supports the contention that *LDLR* is directly regulated by miR-20a.

### Correlation between miR-20a and low-density lipoprotein receptor expression levels in the porcine liver

**Figure 7:**
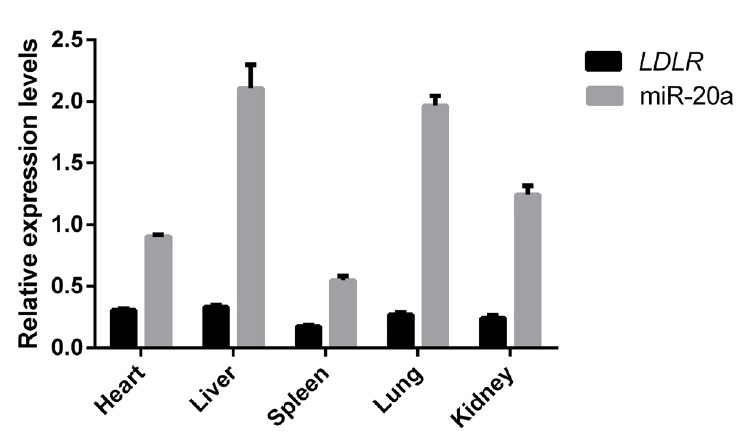
Expression of miR-20a and *LDLR* in different tissues of Large White pigs (n = 12). Expression was determined by RT-qPCR. *LDLR*, low-density lipoprotein receptor; RT-qPCR, reverse transcription quantitative polymerase chain reaction.

Relative expression levels of miR-20a and *LDLR* mRNA in liver, heart, spleen, lung, and kidney tissues of 12 Large White pigs were analyzed using reverse transcription (RT)-qPCR. As shown in Figure 7, *SSC*-miR-20a and *LDLR* were relatively highly expressed in porcine liver tissues. To test whether miR-20a targets *LDLR* mRNA in porcine liver tissues, we analyzed the correlation between these two markers. Pearson correlation analysis revealed that in porcine livers, miR-20a and *LDLR* expression levels were significantly negatively correlated (r = −0.656, p<0.05).

## DISCUSSION

Atherosclerosis is primarily caused by an imbalance of blood lipids, and in particular, the accumulation of LDL-C particles plays a significant role in disease progression [17]. The hepatic LDLR pathway is essential for clearing circulating LDL-C. Whereas the transcriptional regulation of LDLR is well characterized, the post-transcriptional mechanisms that govern its expression are only beginning to emerge [18]. The contribution of miRNAs to the post-transcriptional regulation of *LDLR* expression has recently been demonstrated. Notably, miR-27a/b, miR-185, miR-199a, miR-148a, miR-128-1, miR-130b, and miR-301 were shown to directly target the 3′-UTR of *LDLR* to modulate its expression in human and mouse hepatic cells [19–21]. Recent evidence also suggests that miR-20a contributes to atherosclerosis progression by targeting genes encoding ATP-binding cassette transporter A1 and phosphatase and tensin homolog to regulate their expression [22,23].

In the present study, bioinformatic analysis predicted that *SSC*-miR-20a, which shares 100% and 94.4% nucleotide similarity with human and mouse orthologs, respectively, directly binds porcine *LDLR* mRNA with low free energy of binding. A luciferase-reporter assay confirmed that *SSC*-miR-20a binds a *LDLR* 3′-UTR construct. In addition, *SSC*-miR-20a and *LDLR* were highly expressed in the porcine liver, and liver miR-20a and *LDLR* expression levels were significantly negatively correlated. Together, our findings indicated that *SSC*-miR-20a might act as a negative regulator of porcine *LDLR* expression and protein activity.

The liver is the main site of circulating LDL-C clearance, and LDLR plays a crucial role in hepatic LDL-C uptake [10]. The high expression of *SSC*-miR-20a and *LDLR* in the porcine liver suggested that miR-20a is a novel important regulator of hepatic LDL-C clearance, and functions through the direct regulation of LDLR expression. Bioinformatics predictions and experimental approaches have previously indicated that a single miR can target more than 100 mRNAs, and similarly, one gene can be regulated by multiple miRs [24]. Given the diversity of miRNAs and their targets, the mechanism and function of miR-specific regulation of mRNAs are complicated. However, highly expressed miRs generally have an inhibitory effect on target mRNA expression. In our model (12 Large White castrated boars with body weights of approximately 100 kg), porcine liver miR-20a was found to be negatively correlated with *LDLR* mRNA expression levels; this warrants further, larger studies to confirm the effect of miR-20a on inhibiting LDLR expression.

In conclusion, luciferase activity and RT-qPCR assays suggested that *LDLR* is a target of *SSC*-miR-20a. These results, suggesting that *SSC*-miR-20a can bind *LDLR*, indicate that further study is required to fully elucidate the role of miR-20a in the pathogenesis and progression of cardiovascular diseases.

## Figures and Tables

**Table 1:** Sequences of primers used for reverse transcription quantitative polymerase chain reaction

Target	Accession no.	Primer sequence (5′–3′)
*SSC*-miR-20a	MIMAT0002129	F: TAAAGTGCTTATAGTGCAGGTA
U6 gene	ENSSSCT00000019750	F: GGCAAGGATGACACGCAAAT
*β-Actin*	XM-003124280.2	F: CTCTTCCAGCCCTCCTTCC
		R: GGTCCTTGCGGATGTCG
*LDLR*	NM_001206354.2	F: AGGGTTAGGGGCTCTGAACA
		R: CCCAGCTTGGAAACCCTCAT

U6, porcine U6 small nuclear RNA; *LDLR*, low-density lipoprotein receptor; *SSC*, *Sus scrofa*.

**Table 2:** Sequences of primers used to amplify gene fragments and miRNAs

Target fragment	Primer sequence (5′–3′)
WT *LDLR*	F: AGATCGCCGTGTAATTCTAGATGGTCAGCTTGGAGGATGAC R: GCCGGCCGCCCCGACTCTAGAGGTGAATGAATGGGTCTCAG
MT *LDLR*	F: CGTTTTGGCGCCTCAGTCTC R: GAGACTGAGGCGCCAAAACGACTGTTGGTGGTTACAG
miR-20a	F: CCGGAAACTAGTCTCAGATCTGTGTCGATGTAGAATCTGC R: GATTATGATCAGTTATCTAGACAGTAACTGGACAGTTTGAC

*LDLR*, low-density lipoprotein receptor.
